# FOXM1/DVL2/Snail axis drives metastasis and chemoresistance of colorectal cancer

**DOI:** 10.18632/aging.202300

**Published:** 2020-12-03

**Authors:** Yuhan Yang, Hequn Jiang, Wanxin Li, Linyi Chen, Wanglong Zhu, Yu Xian, Zhengyu Han, Lan Yin, Yao Liu, Yi Wang, Kejian Pan, Kun Zhang

**Affiliations:** 1 School of Bioscience and Technology, Chengdu Medical College, Chengdu, China; 2 First Afflicted Hospital, Chengdu Medical College, Chengdu, China; 3 School of Pharmacy, Chengdu Medical College, Chengdu, China; 4 School of Medical Laboratory Science, Chengdu Medical College, Chengdu, China

**Keywords:** metastasis, chemoresistance, disheveled, colorectal cancer, FOXM1

## Abstract

Colorectal cancer (CRC) is the third most common type of cancer worldwide. Metastasis and chemoresistance are regarded as the two leading causes of treatment failure and high mortality in CRC. Forkhead Box M1 (FOXM1) has been involved in malignant behaviors of cancer. However, the role and mechanism of FOXM1 in simultaneously regulating metastasis and chemoresistance of CRC remain poorly understood. Here, we found that FOXM1 was overexpressed in oxaliplatin- and vincristine-resistant CRC cells (HCT-8/L-OHP and HCT-8/VCR) with enhanced metastatic potential, compared with HCT-8 cells. FOXM1 overexpression increased migration, invasion and drug-resistance to oxaliplatin and vincristine in HCT-8 cells, while FOXM1 knockdown using shFOXM1 impaired metastasis and drug-resistance in HCT-8/L-OHP and HCT-8/VCR cells. Moreover, FOXM1 up-regulated Snail to trigger epithelial-mesenchymal transition-like molecular changes and multidrug-resistance protein P-gp expression, while silencing Snail inhibited FOXM1-induced metastasis and drug-resistance. We further identified that disheveled-2 (DVL2) was crucial for FOXM1-induced Snail expression, metastasis and chemoresistance. Furthermore, FOXM1 bound to DVL2, and enhanced nuclear translocation of DVL2 and DVL2-mediated transcriptional activity of Wnt/β-catenin known to induce Snail expression. In conclusion, FOXM1/DVL2/Snail axis triggered aggressiveness of CRC. Blocking FOXM1/DVL2/Snail pathway simultaneously inhibited metastasis and chemoresistance in CRC cells, providing a new strategy for successful CRC treatment.

## INTRODUCTION

Colorectal cancer (CRC) is reported as the third most frequently diagnosed malignancy, as well as the fourth leading cause of cancer-related mortality worldwide [1, 2]. Although advances in surgical operation and the use of combined systemic drug therapy have contributed to a decrease in the rate of cancer mortality [3, 4], a large number of CRC patients still inexorably experience two persisting challenges, cancer cell metastasis and drug-resistance in the following years [5, 6]. Indeed, these two intractable issues are the major causes of failure in cancer therapy [7, 8], but the underlying mechanism has not been completely elucidated. Therefore, it is necessary to understand the biology of metastasis and chemoresistance for effective improvement in CRC therapy.

As a well-known process in enhancing cell motility, epithelial-mesenchymal transition (EMT) could also help cancer cells promote metastasis [9]. Epithelial cells undergoing EMT lose cell-cell adhesion and cell polarity, and they acquire mesenchymal features to become more migratory and invasive [10]. Recently acquired evidence suggests that EMT not only heightens metastasis of cancer cells but also contributes to chemoresistance [11–14]. Numerous studies have shown that transcription factors, including Snail, Slug, and Twist closely participate in EMT [15]. These transcription factors can down-regulate epithelial marker E-cadherin, while up-regulate the mesenchymal markers such as N-cadherin and Vimentin, promoting the tendency of cell to mesenchymal-like features [16]. Moreover, EMT is initiated and controlled by altered signaling pathways including Wnt signaling which activates Snail expression [16, 17]. Disheveled (DVL) is a key hub that bridges receptors and downstream components of Wnt pathway [18, 19]. Notably, even without Wnt ligand, DVL can potently activate Wnt/β-catenin signaling [20–22]. DVL is also found to be up-regulated in progressive and recurrent cancers [23–26]. However, the effect of DVL on EMT and EMT-mediated metastasis and chemoresistance in CRC remains unclear.

Forkhead box (FOX) proteins are a superfamily of evolutionarily conserved transcription factors [27]. FOXM1, a member of FOX superfamily, is required for proliferation of normal cells and embryonic development [28, 29]. The dysfunction of FOXM1 exists in almost all cancers, and has been implicated in all major hallmarks of cancer defined by Weinberg and Hanahan [30, 31]. Overexpression of FOXM1 can promote cell migration and invasion, and induce premetastatic niche at the distal organ of metastasis in cancer cells [27, 32, 33]. FOXM1 directly activates genes implicated in multiple phases of metastasis, and has been reported as the master regulator of metastasis in breast cancer, pancreatic cancer, melanoma and hepatocellular carcinoma [34, 35]. Moreover, abnormal activation of FOXM1 also contributes to drug-resistance in cancers including ovarian cancer, breast cancer, prostate cancer, nasopharyngeal carcinoma, acute myeloid leukemia and colorectal cancer [36–41]. Inhibiting FOXM1 significantly improves chemosensitivity via suppression of drug efflux pump and promotion of cytotoxic and proapoptotic effects of therapeutics [42, 43]. Recently, it has been suggested that FOXM1-regulatory network is a critical predictor of poor prognosis in 18,000 cancer cases across 39 human malignancies [44]. However, little is known about the underlying mechanism by which FOXM1 simultaneously regulates metastasis and chemoresistance of CRC.

In this study, we found that FOXM1 simultaneously contributed to migration, invasion, and drug-resistance in CRC cells via EMT crucial transcription factor Snail. As a proof, knockdown of Snail abolished FOXM1-regulated expressions of EMT-associated markers and P-gp. Moreover, we confirmed that FOXM1 bound to DVL2 and increased nuclear translocation of DVL2 and DVL2-mediated transcriptional activity of Wnt/β-catenin. Silencing DVL2 reduced FOXM1-mediated Snail expression, metastasis and drug-resistance. In conclusion, our results revealed that FOXM1/DVL2/Snail axis simultaneously conferred metastasis and chemoresistance of CRC, providing a novel strategy for improving CRC therapy.

## RESULTS

### Knockdown of FOXM1 suppressed migration and invasion in drug-resistant CRC cells and promoted chemosensitivity

**Figure 1:**
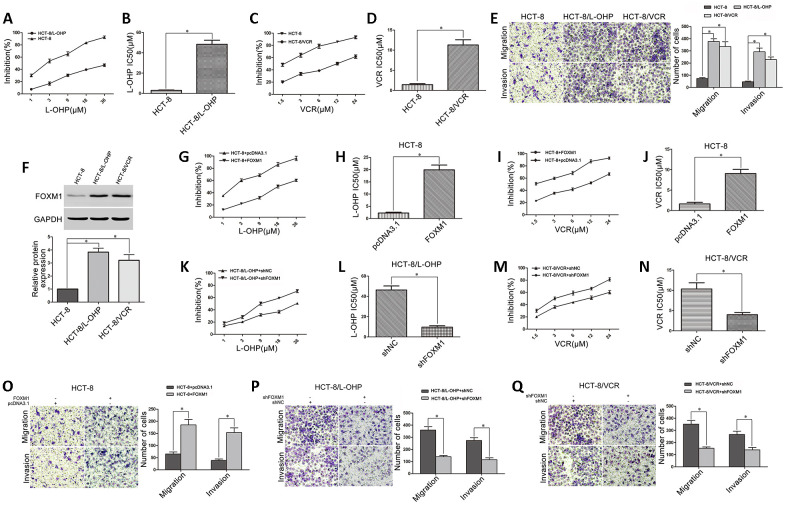
**The role of FOXM1 in metastasis and drug-resistance of CRC cells.** (**A**) The sensitivities of HCT-8 and HCT-8/L-OHP cells to oxaliplatin were assessed using MTT assays. (**B**) The IC50 of oxaliplatin in HCT-8 and HCT-8/L-OHP cells. (**C**) The sensitivities of HCT-8 and HCT-8/VCR cells to vincristine. (**D**) The IC50 of vincristine in HCT-8 and HCT-8/VCR cells. (**E**) The migratory and invasive behaviors of HCT-8, HCT-8/L-OHP and HCT-8/VCR cells were examined using transwell and matrigel invasion assays. (**F**) Western blotting for FOXM1 expression in HCT-8, HCT-8/L-OHP and HCT-8/VCR cells. GAPDH was used as the internal control, and the relative quantitation of FOXM1 expression was normalized against GAPDH using Image J analysis. The oxaliplatin sensitivity and IC50 (**G**, **H**), as well as the vincristine sensitivity and IC50 (**I**, **J**) in HCT-8 cells transfected with pcDNA3.1 or pcDNA3.1-FOXM1. (**K**, **L**) The oxaliplatin sensitivity and IC50 in HCT-8/L-OHP cells transfected with shNC or shFOXM1. (**M**, **N**) The vincristine sensitivity and IC50 in HCT-8/VCR cells transfected with shNC or shFOXM1. The migratory and invasive behaviors were examined using transwell and matrigel invasion assays in HCT-8 cells transfected with pcDNA3.1 or pcDNA3.1-FOXM1 (**O**), in HCT-8/L-OHP and HCT-8/VCR cells transfected with shNC or sh-FOXM1 (**P**, **Q**). Data are expressed as mean ± SD of three independent experiments. *P < 0.05.

The drug-resistant CRC cell models (HCT-8/L-OHP and HCT-8/VCR) were established from the human CRC cell line HCT-8 through serial oxaliplatin (L-OHP) and vincristine (VCR) induction. We identified that HCT-8/L-OHP (IC50 48.39μM) and HCT-8/VCR (IC50 11.29μM) cells were respectively more resistant to oxaliplatin and vincristine than parental HCT-8 cells (IC50 2.98μM; 1.42μM) (Figure 1A–1D). Moreover, we compared cell metastasis between drug-resistant and parental CRC cells. The increased migratory and invasive potential in HCT-8/L-OHP and HCT-8/VCR cells were observed compared with HCT-8 cells (Figure 1E), suggesting that the drug-resistant CRC cells were endowed with enhanced metastasis. Furthermore, FOXM1 was overexpressed in HCT-8/L-OHP and HCT-8/VCR cells compared with HCT-8 cells (Figure 1F), suggesting that FOXM1 was involved in metastasis and drug-resistance of CRC cells.

Next, we determined the possibility of targeting FOXM1 to suppress both metastasis and drug- resistance. The data acquired showed that cell sensitivities to oxaliplatin (IC50 2.25μM *vs* 19.92μM) and vincristine (IC50 1.61μM *vs* 9.04μM) were significantly reduced in HCT-8 cells transfected with FOXM1 recombinant vector (Figure 1G–1J), while the cell sensitivities to oxaliplatin (IC50 46.26μM *vs* 9.63μM) and vincristine (IC50 10.33μM *vs* 3.96μM) were respectively promoted in HCT-8/L-OHP and HCT-8/VCR cells transfected with shFOXM1 (Figure 1K–1N). Besides, the cell migratory and invasive potential were enhanced in HCT-8 cells transfected with FOXM1 recombinant vector (Figure 1O), while impaired in HCT-8/L-OHP and HCT-8/VCR cells transfected with shFOXM1 (Figure 1P–1Q). These data suggested that FOXM1 was positively associated with metastasis and drug sensitivity, silencing FOXM1 simultaneously inhibited metastasis and chemoresistance of CRC.

### FOXM1 regulated the expression of EMT-associated markers

**Figure 2:**
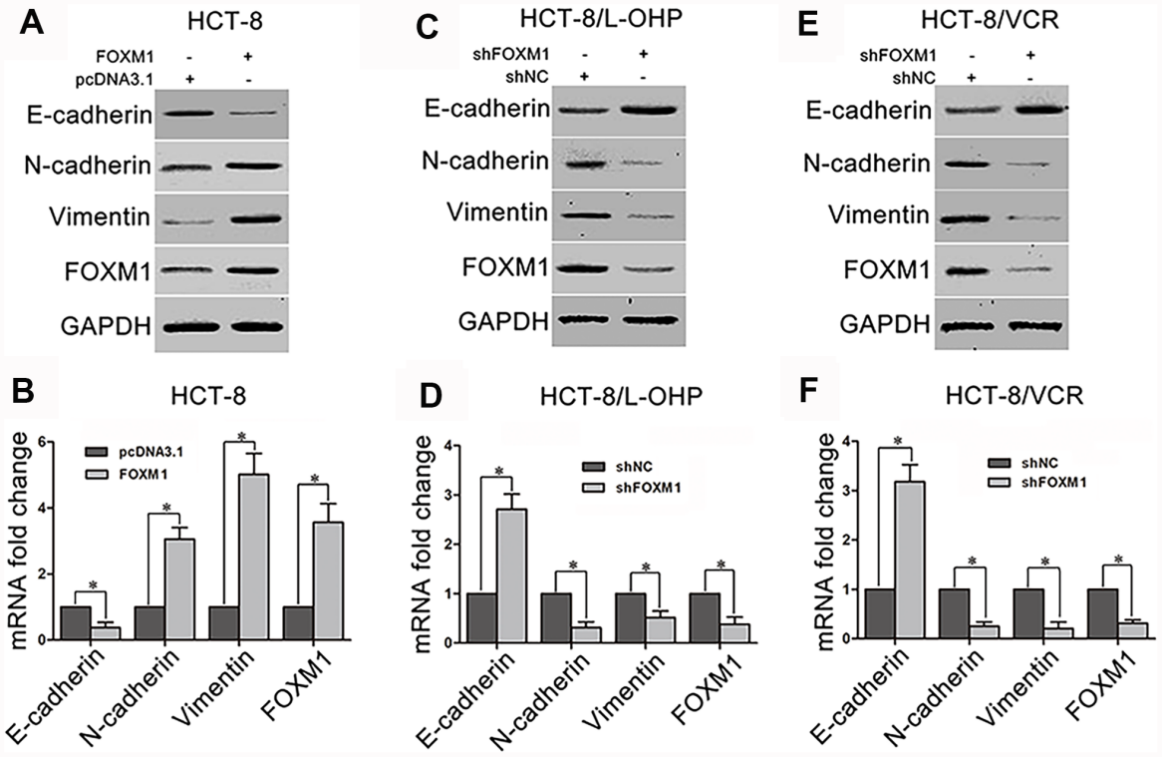
**The effect of FOXM1 on expression of EMT-associated marker.** The protein and mRNA levels of E-cadherin, N-cadherin, Vimentin, and FOXM1 in HCT-8 cells transfected with pcDNA3.1 or pcDNA3.1-FOXM1 for 72 h (**A**, **B**), HCT-8/L-OHP and HCT-8/VCR cells transfected with shNC or shFOXM1 for 72 h (**C–F**). Cell extracts of each sample were prepared and analyzed for protein expression by Western blotting or for mRNA expression by qRT-PCR. For the western blotting, each immunoblot is representative of three separate experiments. For the qRT-PCR, the relative mRNA expression levels were normalized to the fold change that was detected in the corresponding control cells, which was defined as 1.0. Data are expressed as mean ± SD of three independent experiments. *P < 0.05.

To explorer underlying mechanism by which FOXM1 simultaneously induced metastasis and chemoresistance of CRC, we examined the effect of FOXM1 on EMT which facilitates both cell motility and drug-resistance. The recombinant vector of FOXM1 was transfected into HCT-8 cells. The results showed that the protein and mRNA levels of epithelial marker E-cadherin were decreased by the ectopic expression of FOXM1 compared with the control group, while the mesenchymal markers N-cadherin and Vimentin were up-regulated (Figure 2A–2B). To validate that the expressions of E-cadherin, N-cadherin and Vimentin were controlled by FOXM1, shFOXM1 was transfected into HCT-8/L-OHP and HCT-8/VCR cells. As predicted, E-cadherin was up-regulated by the knockdown of FOXM1, while N-cadherin and Vimentin were down-regulated (Figure 2C–2F). These results suggested that FOXM1 induced EMT-like molecular changes in CRC cells.

### FOXM1 up-regulated snail to mediate EMT- like molecular changes and P-gp expression

**Figure 3:**
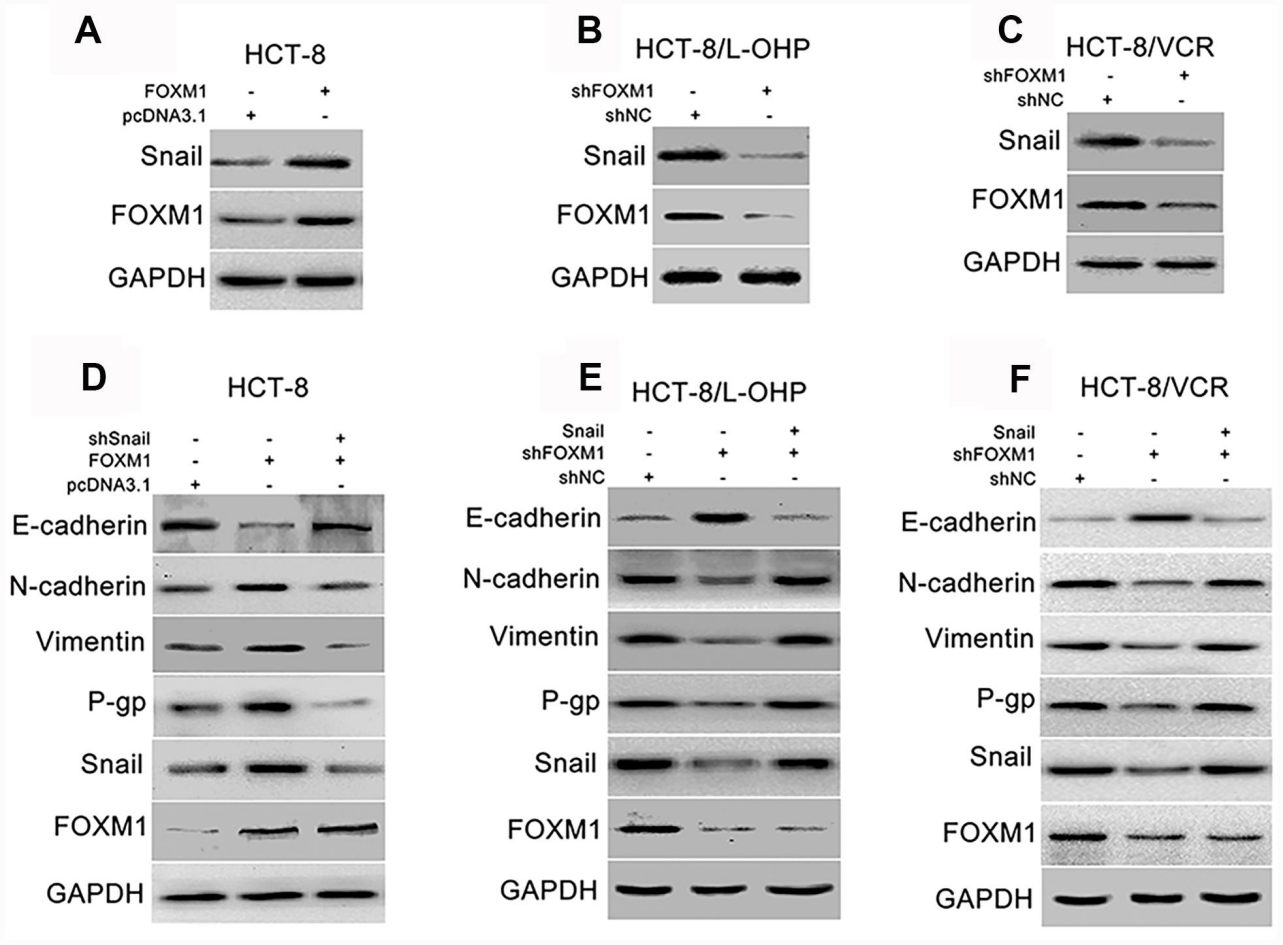
**FOXM1 triggered EMT-like molecular changes and P-gp expression via up-regulating Snail.** Western blotting for expressions of Snail and FOXM1 in HCT-8 cells transfected with pcDNA3.1 or pcDNA3.1-FOXM1 for 72 h (**A**), HCT-8/L-OHP and HCT-8/VCR cells transfected with shNC or shFOXM1 for 72 h (**B**, **C**). The expressions of E-cadherin, N-cadherin, Vimentin, P-gp, Snail and FOXM1 in HCT-8 cells transfected with pcDNA3.1, pcDNA3.1-FOXM1, or pcDNA3.1-FOXM1 plus shSnail for 72 h (**D**), HCT-8/L-OHP and HCT-8/VCR cells transfected with shNC, shFOXM1, or shFOXM1 plus pcDNA3.1-Snail (**E**, **F**). In each case, the blot is representative of immunoblots resulting from three separate experiments.

Snail is regarded as the core transcription factor of EMT [45, 46]. So, we evaluated the effect of FOXM1 on Snail, and the role of Snail in FOXM1-mediated expression of EMT-associated markers. The protein level of Snail was increased by overexpression of FOXM1 in HCT-8 cells transfected with recombinant vectors of FOXM1 (Figure 3A), while reduced by knockdown of FOXM1 in HCT-8/L-OHP and HCT-8/VCR cells transfected with shFOXM1 (Figure 3B–3C). Next, the recombinant vector of FOXM1 was co-transfected with shSnail into HCT-8 cells. Our results showed that FOXM1-induced down-regulation of E-cadherin, up-regulation of N-cadherin and Vimentin were eliminated by silencing Snail (Figure 3D). Moreover, the recombinant vector of Snail was co-transfected with shFOXM1 into HCT-8/L-OHP and HCT-8/VCR cells. We found that shFOXM1-induced up-regulation of E-cadherin, down-regulation of N-cadherin and Vimentin were reversed by Snail overexpression (Figure 3E, 3F). Considering that EMT also plays a crucial role in chemoresistance of cancers [12, 13], we further examined the effect of Snail on FOXM1-mediated expression of multidrug-resistance protein P-gp. The results showed that the protein level of P-gp was positively regulated by FOXM1, while FOXM1-induced P-gp expression was abolished by silencing Snail (Figure 3D). In addition, shFOXM1-decreased P-gp expression was rescued by Snail overexpression (Figure 3E, 3F). These data suggested that FOXM1 up-regulated Snail to induce EMT- like molecular changes and P-gp expression.

### FOXM1 triggered metastasis and chemoresistance of CRC via Snail

**Figure 4:**
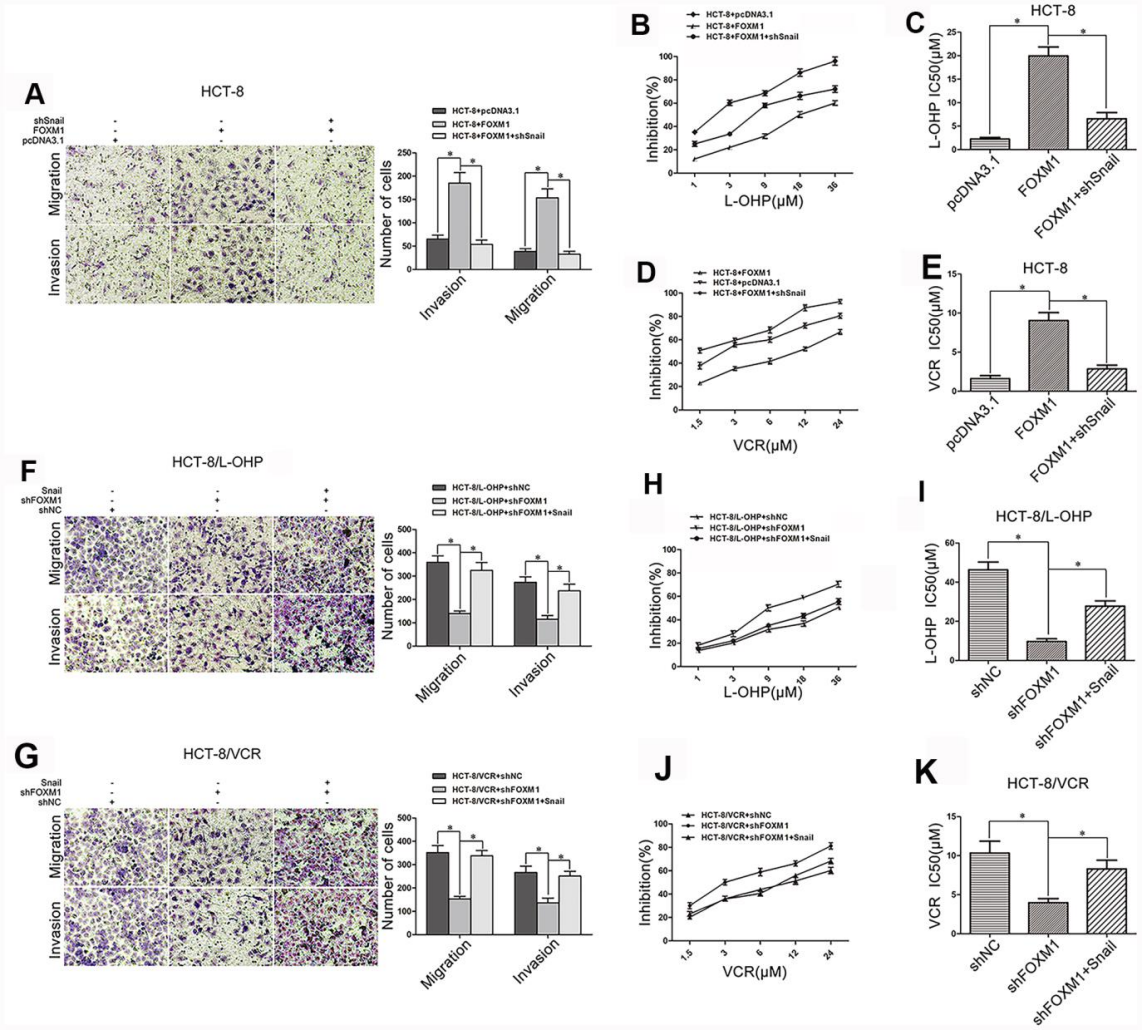
**FOXM1 induced metastasis and chemoresistance of CRC via Snail.** Transwell and matrigel invasion assays for migratory and invasive behaviors (**A**), and MTT assay for oxaliplatin (**B**, **C**) and vincristine (**D**, **E**) sensitivity and IC50 in HCT-8 cells transfected with pcDNA3.1, pcDNA3.1-FOXM1, or pcDNA3.1-FOXM1 plus shSnail for 72 h, as indicated. Transwell and matrigel invasion assays for migratory and invasive behaviors (**F**, **G**), and MTT assay for oxaliplatin (**H**, **I**) or vincristine (**J**, **K**) sensitivity and IC50 in HCT-8/L-OHP or HCT-8/VCR cells transfected with shNC, shFOXM1, or shFOXM1 plus pcDNA3.1-Snail for 72 h, as indicated. Data are expressed as mean ± SD of three independent experiments. *P < 0.05.

We further observed the role of Snail in FOXM1-induced metastasis and chemoresistance of CRC. FOXM1-induced migration and invasion were suppressed by silencing Snail in HCT-8 cells (Figure 4A), and FOXM1-increased drug-resistance to oxaliplatin (IC50 19.92μM *vs* 6.58μM) and vincristine (IC50 9.04 μM *vs* 2.86 μM) were impaired by silencing Snail (Figure 4B–4E). Moreover, shFOXM1-inhibited migration and invasion were rescued by overexpression of Snail in HCT-8/L-OHP and HCT-8/VCR cells (Figure 4F–4G), and shFOXM1-decreased drug-resistance to oxaliplatin (IC50 9.63μM *vs* 27.73μM) and vincristine (IC50 3.96μM *vs* 8.30 μM) were restored by overexpression of Snail (Figure 4H–4K). Above results suggested that FOXM1 induced metastasis and chemoresistance of CRC via Snail.

### DVL2 was critical for FOXM1-induced Snail expression, metastasis, and chemoresistance

**Figure 5:**
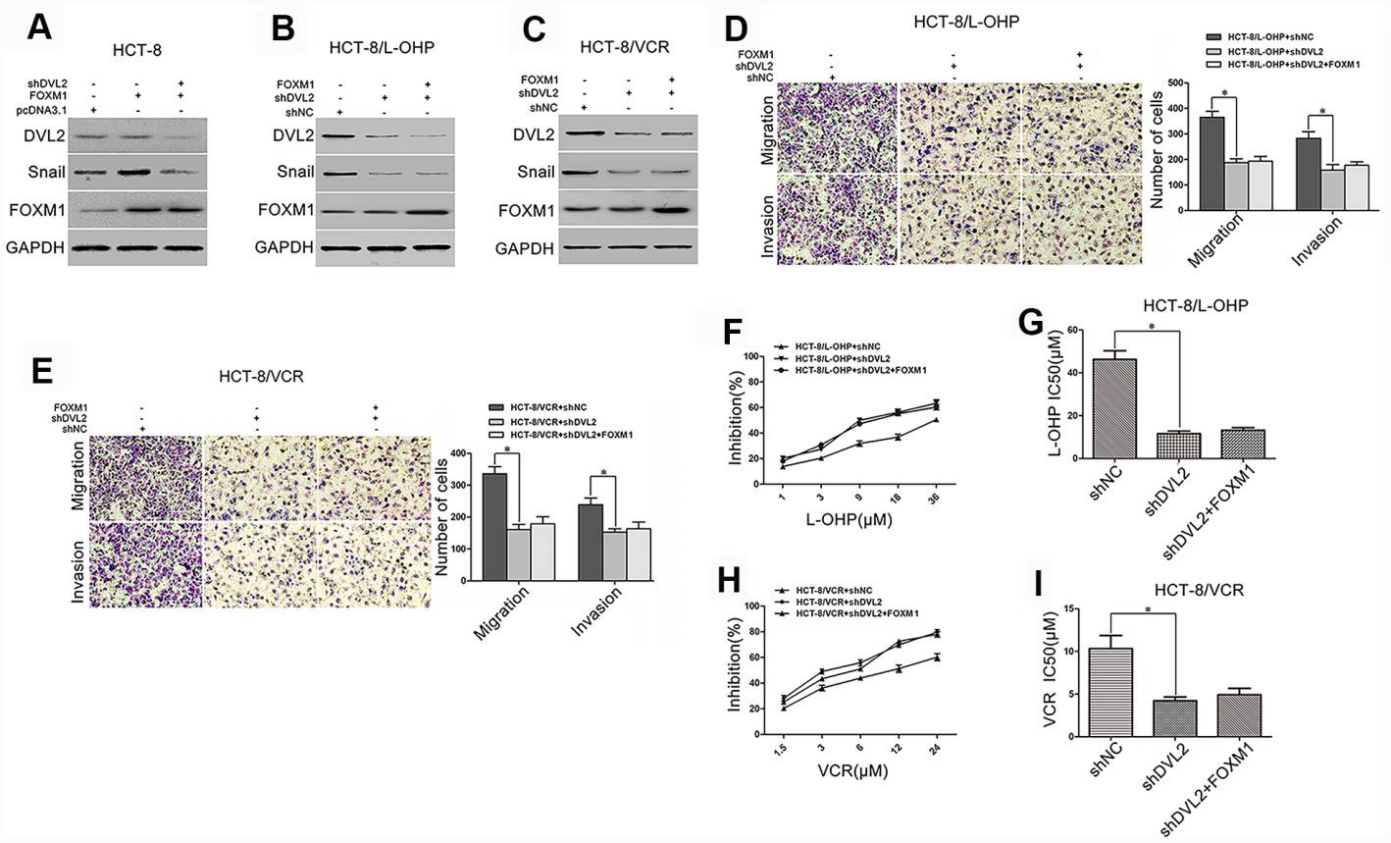
**DVL2 was crucial for FOXM1-mediated Snail expression, metastasis, and chemoresistance.** Western blotting for the expressions of DVL2, Snail and FOXM1 in HCT-8 cells transfected with pcDNA3.1, pcDNA3.1-FOXM1, or pcDNA3.1-FOXM1 plus shDVL2 for 72 h (**A**), HCT-8/L-OHP and HCT-8/VCR cells transfected with shNC, shDVL2, or shDVL2 plus pcDNA3.1-FOXM1 for 72 h (**B**, **C**) In each case, the blot is representative of immunoblots resulting from three separate experiments. Transwell and matrigel invasion assays for migratory and invasive behaviors (**D**, **E**), and MTT assay for oxaliplatin (**F**, **G**) or vincristine (**H**, **I**) sensitivity and IC50 in HCT-8/L-OHP or HCT-8/VCR cells transfected with shNC, shDVL2, or shDVL2 plus pcDNA3.1-FOXM1 for 72 h, as indicated. Data are expressed as mean ± SD of three independent experiments. *P < 0.05.

Although we have identified that FOXM1 induced the expression of Snail, this was not the case in DVL2 knockdown HCT-8 cells (Figure 5A). It was sure that Snail expression was decreased by silencing DVL2 in HCT-8/L-OHP and HCT-8/VCR cells. When DVL2 was silenced by shDVL2, the decreased Snail protein level was not rescued by FOXM1 (Figure 5B–5C). Meanwhile, the effect of FOXM1 on expression of DVL2 was observed. Unexpectedly, the expression of DVL2 was not significantly changed by ectopic expression of FOXM1 in HCT-8, HCT-8/L-OHP and HCT-8/VCR cells, and the down-regulation of DVL2 by shDVL2 was not rescued by FOXM1 (Figure 5A–5C). These results suggested that FOXM1 induced Snail expression via DVL2, independently of DVL2 expression. Furthermore, the migration and invasion, and drug-resistance to oxaliplatin (IC50 46.26 μM *vs* 11.56 μM) and vincristine (IC50 10.33 μM *vs* 4.21 μM) were inhibited by silencing DVL2 in HCT-8/L-OHP and HCT-8/VCR cells (Figure 5D–5I). Meanwhile, FOXM1 lost the ability to induce migration, invasion and drug-resistance when DVL2 was silenced (Figure 5D–5I). Collectively, above data indicated that DVL2 was required for FOXM1-mediated Snail expression, metastasis and chemoresistance, while FOXM1 did not change expression of DVL2.

### FOXM1 bound to DVL2 and promoted nuclear translocation of DVL2

**Figure 6:**
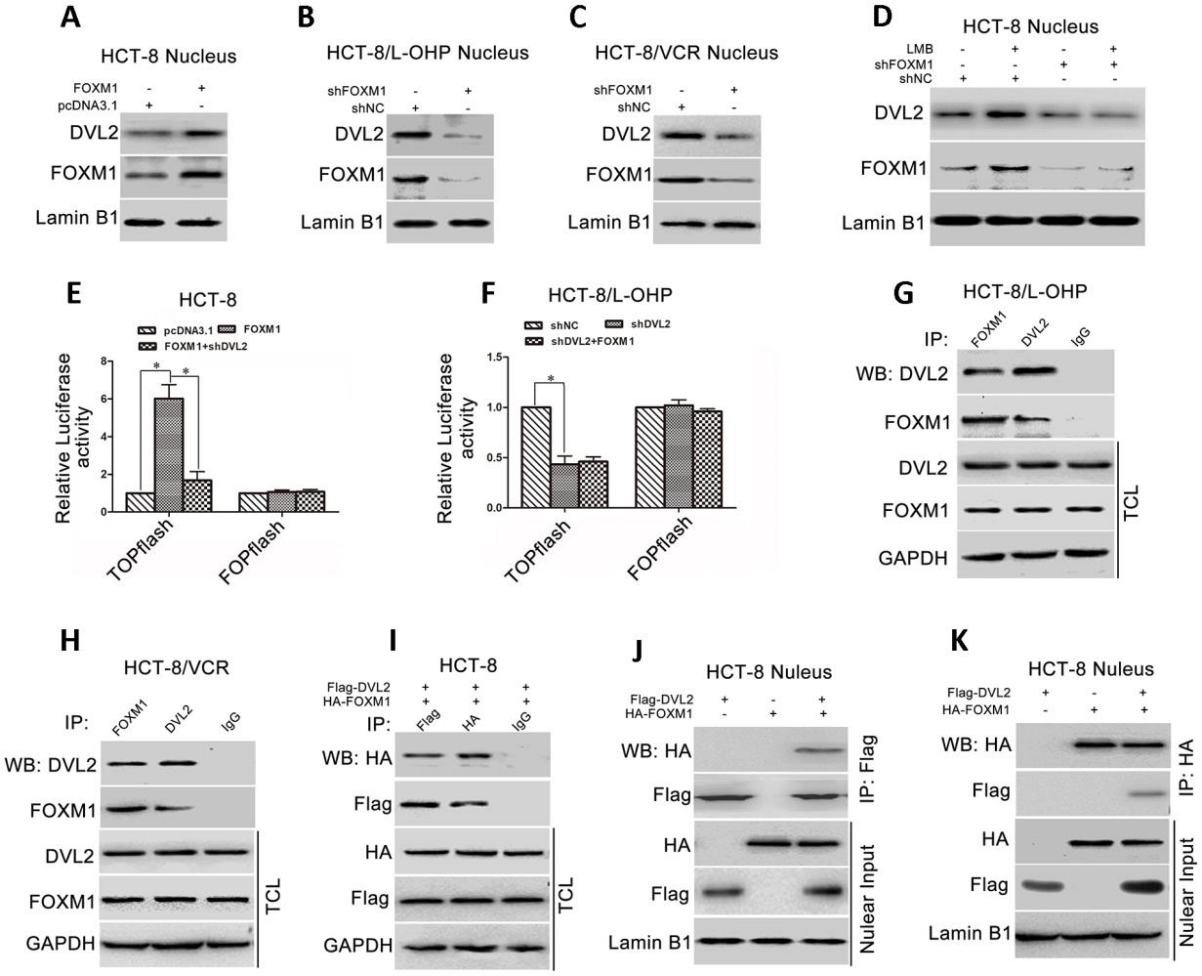
**FOXM1 bound to DVL2 and enhanced its nuclear translocation.** Western blotting for the nuclear DVL2 and FOXM1 expressions in HCT-8 cells transfected with pcDNA3.1 or pcDNA3.1-FOXM1 (**A**), HCT-8/L-OHP and HCT-8/VCR cells transfected with shNC or shFOXM1 (**B**, **C**), HCT-8 cells treated with or without 50 ng/ml LMB for 12 h in presence of shNC or shFOXM1 transfection for 72 h (**D**), as indicated. Dual-luciferase reporter assay for TOPflash and FOPflash luciferase activity in HCT-8 cells transfected with pcDNA3.1, pcDNA3.1-FOXM1, or pcDNA3.1-FOXM1 plus shDVL2 for 48 h (**E**), HCT-8/L-OHP cells transfected with shNC, shDVL2, or shDVL2 plus pcDNA3.1-FOXM1 for 48 h (**F**). The relative luciferase activity was normalized against Renilla reporter pRL-SV40 activity. Coimmunoprecipitation (Co-IP) of endogenous FOXM1 and DVL2 in HCT-8/L-OHP cells and HCT-8/VCR cells (**G**, **H**). Co-IP of fusion protein HA-FOXM1 and Flag-DVL2 in total cellular lysates (TCL) and nuclear fractions in HCT-8 cells transfected with pcDNA3.1-HA-FOXM1 and/or pcDNA3.1-Flag-DVL2 for 72 h (**I**–**K**). In each case, the blot is representative of immunoblots resulting from three separate experiments. Data are expressed as mean ± SD of three independent experiments. *P < 0.05.

Accumulating evidence has confirmed that nuclear translocation of DVL plays a critical role in Wnt/β-catenin signaling that can activate Snail expression [47–50]. To understand how FOXM1 promoted Snail expression via DVL2, we examined the effect of FOXM1 on nuclear distribution of DVL2 in CRC cells. The results showed that the protein level of nuclear DVL2 was increased by ectopic expression of FOXM1 in HCT-8 cells (Figure 6A), while reduced by knockdown of FOXM1 in HCT-8/L-OHP and HCT-8/VCR cells (Figure 6B, 6C). Moreover, leptomycin B (LMB), an inhibitor of nuclear export machinery, was used to treat the cells. The results showed that LMB increased nuclear accumulation of DVL2 in HCT-8 cells, but not in the FOXM1 knockdown cells (Figure 6D). These data suggested that FOXM1 enhanced nuclear translocation of DVL2 in CRC cells.

We next examined whether FOXM1 controlled DVL2-mediated transcription activity of Wnt/β-catenin. TOPflash and FOPflash luciferase reporters, which respectively contain the wildtype and mutant β-catenin/TCF-binding site, were widely employed to characterize β-catenin/TCF transcription activity in nucleus [51]. Dual-luciferase reporter assay showed that TOPflash luciferase activity was higher in HCT-8 cells transfected with recombinant vector of FOXM1 relative to control group (6.02 fold) (Figure 6A), while lower in HCT-8 cells co-transfected with shDVL2 and recombinant vector of FOXM1 (72.13% reduction) (Figure 6E). It was sure that TOPflash luciferase activity was lower in HCT-8/L-OHP cells transfected with shDVL2 relative to control group (56.67% reduction). However, FOXM1 cannot restore TOPflash luciferase activity in HCT-8/L-OHP cells transfected with shDVL2 (Figure 6F). Meanwhile, after ectopic expression of FOXM1 or silencing DVL2, no significant difference was observed in FOPflash luciferase activity compared with control cells (Figure 6E, 6F). These data suggested that FOXM1 enhanced nuclear translocation of DVL2 and DVL2-mediated transcriptional activity of Wnt/β-catenin.

We further explored how FOXM1 regulated the nuclear accumulation of DVL2. The physical association between FOXM1 and DVL2 was checked. The results of co-immunoprecipitation (Co-IP) revealed that endogenous FOXM1 and DVL2 were precipitated down by each other in HCT-8/L-OHP and HCT-8/VCR cells (Figure 6G, 6H). Furthermore, the recombinant vector of HA-FOXM1 and Flag-DVL2 were transfected into HCT-8 cells. The fusion protein HA-FOXM1 was physically associated with Flag-DVL2 in the nucleus (Figure 6I–6K). Collectively, above data suggested that FOXM1 bound to DVL2 and facilitated nuclear translocation of DVL2 and DVL2-mediated transcriptional activity of Wnt/β-catenin.

## DISCUSSION

Colorectal cancer (CRC) is an extensive solid malignancy and the fourth leading cause of cancer mortality worldwide [52], which deserves intensive investigation. In clinical practice, a lack of sensitivity to chemotherapy can be estimated on the basis of the modified Response Evaluation Criteria in Solid Tumors (mRIST) criteria, and this lack of sensitivity can be connected with another round of uncontrollable proliferation or even metastasis [53]. Increasing evidence has revealed that chemoresistance and metastasis are closely linked phenotypes during progression of malignancy [54, 55]. In this study, we identified that oxaliplatin-resistant and vincristine-resistant CRC cells (HCT-8/L-OHP and HCT-8/VCR), the universal cell models for analyzing acquired chemoresistance in CRC, were endowed with enhanced migratory and invasive capacities (Figure 1A–1E). It may be an underlying reason that a considerable proportion of CRC patients with adjuvant chemotherapy still suffer distant metastasis. Indeed, metastasis and chemoresistance have be regarded as the primary barriers to successful cancer therapy [56]. Therefore, it is urgent to explore mechanism responsible for metastasis and chemoresistance in CRC and develop efficient strategy to improve therapeutic efficacy.

Our present study showed that FOXM1 was overexpressed in HCT-8/L-OHP and HCT-8/VCR cells marked by enhanced migration and invasion (Figure 1F), suggesting that FOXM1 was implicated in metastasis and chemoresistance of CRC. Although earlier studies have showed that FOXM1 contributes to the progression of malignancy in multiple tumor types [57], the mechanism by which FOXM1 simultaneously triggers metastasis and chemoresistance remains poorly understood in CRC. Here, our acquired data showed that ectopic expression of FOXM1 not only promoted drug-resistance to oxaliplatin and vincristine in HCT-8 cells but also increased the metastatic potential, while knockdown of FOXM1 impaired drug-resistance and metastasis in HCT-8/L-OHP and HCT-8/VCR cells (Figure 1G–1Q), suggesting that FOXM1 could drive both metastasis and chemoresistance of CRC. Remarkably, accumulating evidence suggests that EMT might be a process that initiates both cancer metastasis and chemoresistance in different cancers, including lung cancer and hepatocellular carcinoma [58, 59]. EMT is characterized by the loss of epithelial markers and acquisition of mesenchymal markers [16]. Our data showed that FOXM1 induced EMT-like molecular changes where the epithelial marker E-cadherin was decreased while the mesenchymal markers N-cadherin and vimentin were up-regulated (Figure 2). These results suggested that EMT was associated with FOXM1-driven metastasis and chemoresistance.

Snail is considered to be the core transcription factor that drives EMT of epithelial tumor cells, which is almost involved in the whole process of tumor EMT [45, 46, 60, 61]. Thus, we evaluated the effect of FOXM1 on Snail expression, and the role of Snail in FOXM1-regulated expression of EMT-associated markers. Our results showed that FOXM1 positively regulated Snail expression in HCT-8, HCT-8/L-OHP, and HCT-8/VCR cells (Figure 3A–3C), while silencing Snail eliminated FOXM1-induced EMT-like molecular changes (Figure 3D). Besides, Snail overexpression reversed shFOXM1-mediated EMT-associated markers changes (Figure 3E, 3F). These data indicated that FOXM1 increased Snail expression to trigger EMT-like molecular changes. A substantial body of evidence has confirmed that during EMT, Snail can inhibit epithelial markers and up-regulate mesenchymal markers to reduce cell-cell adhesion and promote cell motility. Elevated Snail expression indicates a high risk of distant metastases of cancer cells [62]. Moreover, Snail also factors into drug-resistance in ovarian, breast, prostate, and head and neck cancers [63–66], although the underlying mechanism is not entirely clear. Interestingly, our data showed that FOXM1 triggered multidrug-resistance protein P-gp expression via Snail in CRC cells (Figure 3D–3F). Collectively, these results suggested that FOXM1 could simultaneously drive metastasis and chemoresistance in CRC cells via Snail. As expected, silencing Snail suppressed FOXM1-induced migration, invasion, and drug-resistance to oxaliplatin and vincristine in HCT-8 cells (Figure 4A–4E), while overexpression of Snail rescued shFOXM1-dereased migration, invasion, and drug-resistance in HCT-8/L-OHP and HCT-8/VCR cells (Figure 3F–3K).

We then focused on the potential mechanism by which FOXM1 up-regulated Snail. Recent reports have shown that Snail expression was controlled by multiple signaling including Wnt and TGF-β [16, 67]. Disheveled (DVL) is known as the hub of Wnt signaling, which transmits Wnt signals from receptors to downstream effectors [18]. Not only that, DVL also bridges cross-talk between Wnt and other signaling including TGF-β [68, 69]. Therefore, the role of DVL in FOXM1-induced expression of Snail was assessed. Our data showed that silencing DVL2 abolished FOXM1-increased Snail expression in HCT-8 cells, and FOXM1 lost the ability to up-regulate Snail when DVL2 was silenced in HCT-8/L-OHP and HCT-8/VCR cells (Figure 5A–5C), suggesting that FOXM1 increased Snail expression via DVL2. Notably, even without stimulation of Wnt ligand, the ectopic expression of DVL can be sufficient to activate Wnt/β-catenin which induced Snail expression [20, 21, 67, 70]. Therefore, we envisioned that FOXM1 promoted DVL2 expression to up-regulate Snail. However, our hypothesis was not supported by the result revealing that FOXM1 did not significantly affect the protein level of DVL2 (Figure 5A–5C). These results indicated that FOXM1 up-regulated Snail expression via DVL2, independently of DVL2 expression. Furthermore, silencing DVL2 decreased metastasis and drug-resistance in HCT-8/L-OHP and HCT-8/VCR cells (Figure 5D–5I). Although our preliminary results have revealed that FOXM1 induced metastasis and drug-resistance of CRC cells (Figure 1G–1Q), this was not the case in DVL2 silencing CRC cells (Figure 5D–5I). Collectively, above results indicated that DVL2 was required for FOXM1-induced Snail expression, metastasis and chemoresistance, while FOXM1 did not change expression of DVL2.

Previous studies have revealed that DVL can shuttle between cytoplasm and nucleus, nuclear localization of DVL is pivotal for its function in Wnt signaling [71, 72]. Nuclear DVL acts as a coactivator to enhance activity of β-catenin/TCF-4 [49, 70]. In addition, Wnt/β-catenin signaling has been shown to activate Snail expression and enhance its protein stability [16, 67, 73, 74]. Therefore, to further explore how FOXM1 induced Snail expression via DVL2, the effect of FOXM1 on nuclear accumulation of DVL2 was assessed. The results showed that FOXM1 promoted nuclear translocation of DVL2 in HCT-8, HCT-8/L-OHP and HCT-8/VCR cells (Figure 6A–6C). Moreover, inhibition of nuclear export machinery using leptomycin B (LMB) enhanced nuclear accumulation of DVL2. However, this case was not observed in FOXM1 silencing cells (Figure 6D). These data suggested that FOXM1 promoted nuclear translocation of DVL2 in CRC cells. Increasing evidence has shown that although β-catenin can bind to TCFs, this bilateral interaction is not enough, albeit necessary, for activation of Wnt/β-catenin which requires binding of coactivators, such as BCl9 and Pygopus, to β-catenin in nucleus [75, 76]. In fact, the interaction of nuclear DVL with β-catenin has been also observed and found to facilitate the transcription complex formation of β-catenin/TCF or its stability on the promoter of Wnt target genes [49]. These results reveal the transcriptional function of DVL. Our results showed that silencing DVL2 inhibited FOXM1-increased β-catenin/TCF-4 transcriptional activity in CRC cells, while FOXM1 did not significantly promote the activation of β-catenin/TCF-4 when DVL2 was silenced (Figure 6E, 6F). Collectively, these results suggested that FOXM1 enhanced nuclear translocation of DVL2 to promote DVL2-mediated transcriptional activity of Wnt/β-catenin, which increased Snail expression. However, it remains unclear how FOXM1 promoted nuclear translocation of DVL2. As an important member of Forkhead Box transcription factor family, it has been postulated that the function of FOXM1 is determined by its capacity to transactivate various target genes that are involved in multiple stages of cancer development [30]. However, the latest research suggested that FOXM1 might also act as an oncogene by interacting with other proteins, thus activating different oncogenic signaling pathways [77]. Therefore, we hypothesized physical association between FOXM1 and DVL2. This hypothesis was confirmed by the results revealing that endogenous FOXM1 was coimmunoprecipitated with DVL2 in HCT-8/L-OHP and HCT-8/VCR cells, and the fusion proteins HA-FOXM1 bound to Flag-DVL2 in the nucleus of HCT-8 cells (Figure 6G–6K). Collectively, above results suggested that FOXM1 bound to DVL2 and enhanced nuclear translocation of DVL2 to augment DVL2-mediated transcriptional activity.

In conclusion, this study has presented evidence that FOXM1/DVL2/Snail axis confers aggressiveness of CRC. FOXM1 bound to DVL2 and facilitated nuclear translocation of DVL2 to promote DVL2-mediated transcriptional activity, Snail expression, and induced EMT-like molecular changes and multidrug-resistance protein P-gp expression which are simultaneously executed by Snail, resulting in both metastasis and chemoresistance in CRC cells. FOXM1/DVL2/Snail axis might be a potential therapeutic target of CRC, especially for the CRC patients who have simultaneously developed metastasis and chemoresistance, supplying a new strategy for successful CRC treatment.

## MATERIALS AND METHODS

### Reagents and antibodies

Oxaliplatin (L-OHP) and vincristine (VCR) were purchased from Selleck Chemicals (Houston, TX, USA). Fetal bovine serum (FBS), RPMI-1640 culture medium, penicillin, and streptomycin were purchased from Gibco (Carlsbad, CA, USA). shFOXM1 (target sequence, 5'- CTCTTCTCCCTCAGATATA-3'), shDVL2 (target sequence, 5'-GGAAGAAATTTCAGATGAC-3'), shSnail (target sequence, 5'- GCCTTCAACTGCAAATACT-3'), and shRNA negative control (shNC) were gained from Genechem (Shanghai, China). cDNAs encoding FOXM1 or Snail was respectively cloned into pcDNA3.1 or pcDNA3.1-HA. pcDNA3.1-Flag-DVL2 was derived from pCMV5-3XFlag-DVL2 which was a gift from Jeff Wrana (Addgene plasmid # 24802) [78]. Lipofectamine 3000 transfection reagent was purchased from Invitrogen (Carlsbad, CA, USA). BCA protein assay kit, Radioimmunoprecipitation (RIPA) lysis buffer, Nuclear and Cytoplasmic Protein Extraction kit, and protein A+G agarose beads were obtained from Beyotime Biotechnology (Nantong, China). Primary antibodies against FOXM1, E-cadherin, N-cadherin, Vimentin, Snail and P-gp were purchased from Cell Signaling Technology (Danvers, MA, USA). Primary antibodies against DVL2, HA, Flag, GAPDH and Lamin B1 were purchased from Santa Cruz Biotechnology (Santa Cruz, CA, USA). The horseradish peroxidase (HRP)-conjugated secondary antibody was purchased from ZSGB-bio (Peking, China).

### Cell culture

HCT-8 colorectal cancer cells (Shanghai Bogoo Biotechnology, Shanghai, China), and their oxaliplatin- and vincristine-resistant derivative cells (HCT-8/L-OHP and HCT-8/VCR) were cultured in RPMI-1640 supplemented with 10% FBS, 100 U/mL penicillin, and 100 μg/mL streptomycin in a humidified 5% CO_2_ incubator at 37° C. To maintain the drug-resistance phenotype, HCT-8/L-OHP and HCT-8/VCR cells were respectively cultured in the presence of 2 μM oxaliplatin and 0.5 μM vincristine sulfate. When cells reached 80‒90% confluency, they were detached with 0.25% Trypsin and then passaged. All cell lines were authenticated by STR profiling before the experiments.

### MTT assay

MTT assays were performed to assess sensitivity of cells to anti-cancer drug. Briefly, cells were seeded in a 96-well plate (5×10^3^/well). 24 h after seeding, the indicated concentrations of oxaliplatin and vincristine were added to cells for 48 h of incubation. Then MTT dye solution (Beyotime Biotechnology, Nantong, China) was added to each well at final concentration of 0.5 mg/mL and incubated for an additional 4 h at 37° C. Following the culture medium was discarded, 150 μl of DMSO (Beyotime Biotechnology, Nantong, China) was added into each well to dissolve formazan blue. The absorbance was measured at 490 nm using an Ultra Microplate Reader (Bio-Tek Instruments, Winooski, USA). Cell viability was expressed as a percentage of the absorbance value of control cultures. Oxaliplatin and vincristine concentrations that achieved 50% growth inhibition (IC50) were calculated from survival curves using the Bliss method.

### Migration and invasion assays

Cell migration was determined using the transwell assay. Briefly, 2.5× 10^4^ cells were resuspended in RPMI-1640 without serum, and seeded in the upper chamber of each transwell (Corning, New York, NY, USA). Then RPMI-1640 with 10% FBS was placed in the lower chamber of each well. Cells were incubated for 24 h at 37° C in a humidified 5% CO_2_ atmosphere. Cells on the top of the filter were removed by wiping with a cotton swab, and the cells that located on the lower surface of filter were fixed and stained with crystal violet (1% in methyl alcohol) for 10 min, followed by cell count. The cell invasion assay was performed similarly, except that the matrigel (BD Biosciences, San Jose, CA, USA) was placed in each well for 6 h before cells were seeded in the upper chamber. After 48 h seeding, matrigel and residual cells in the upper chamber were discard by cotton swabs. The cells on the lower surface of filter were fixed and stained as described above.

### Western blotting

Total proteins were extracted using RIPA lysis buffer. The cytoplasmic and nuclear proteins were extracted using Cytoplasmic Protein Extraction kit. Protein concentration was examined using BCA protein assay kit. Equal amounts of proteins (100 μg/lane) were separated by 10% SDS-PAGE and then transferred onto PVDF membranes (Bio-Rad, Hercules, CA, USA). After blocking with 5% skim milk in phosphate buffer solution (PBS) for 2 h at room temperature, the membranes were incubated with primary antibodies prepared in blocking buffer at 4° C overnight. The next day, the membranes were washed three times with PBS and incubated for 2 h at room temperature with HRP-conjugated secondary antibodies. The membranes were washed three times and protein bands were visualized with an enhanced chemiluminescence detection kit (Invitrogen, Carlsbad, CA, USA) and a Bio-Rad Molecular Imager (Hercules, CA, USA). A mouse monoclonal anti-GAPDH antibody was used as the control for each sample.

### Cell transfection

Cells were seeded in 6- or 96-well plates overnight. The confluent cells (70%–90%) were transfected with pcDNA3.1, pcDNA3.1-FOXM1, pcDNA3.1-HA-FOXM1, pcDNA3.1-Snail, pcDNA3.1-Flag-DVL2, shNC, shFOXM1, shSnail, or shDVL2 using Lipofectamine 3000 transfection reagent according to the manufacturer’s instructions. 24 h or 72 h after transfection, cells were collected and analyzed by the MTT assay, Western blotting, Migration and invasion assays, or Real-time PCR.

### Real-time PCR (qRT-PCR)

Total RNA was extracted using TRIzol (Invitrogen, Carlsbad, CA, USA) from cells and reversely transcribed using the PrimeScript™ RT Reagent Kit (TaKaRa Biotechnology, Tokyo, Japan). Quantitative PCR was performed in an iCycler IQ real-time PCR Detection System (Bio-Rad, Hercules, CA, USA), The relative expression levels were normalized to the fold change that was detected in the corresponding control cells, which was defined as 1.0. The primers (BGI, Shenzhen, china) used in each reaction were as follows: FOXM1 sense primer 5′-AAGCGAGTCCGCATTGCCCC-3′ and antisense primer 5′-CGGGAGGGCCACTTCCA-3′; E-cadherin sense primer 5′-TACACTGCCCAGGAGCCAGA-3′ and antisense primer 5′-TGGCACCAGTGTCCGGATTA-3′; Vimentin sense primer 5′-TGAGTACCGGAGACAGGTGCAG-3′ and antisense primer 5′-TAGCAGCTTCAACGGCAAAGTTC-3′; N-cadherin sense primer 5′-CGAATGGATGAAAGACCCATCC-3′ and antisense primer 5′-GGAGCCACTGCCTTCATAGTCAA-3′; GAPDH sense primer 5′-AGGTGACACTATAGAATAAAGGTGAAGGTCGGAGTCAA-3′ and antisense primer 5′-GTACGACTCACTATAGGGAGATCTCGCTCCTGGAAGATG-3′.

### Luciferase reporter assay

TOPflash and FOPflash reporters (Upstate Biotechnology, Lake Placid, NY, USA) are generally applied to estimate β-catenin/TCF transcriptional activity. TOPflash driven by thymidine kinase promoter contains SIX wildtype β-catenin/TCF-binding sites upstream of a luciferase reporter gene. FOPflash contains SIX mutated β-catenin/TCF-binding sites [51]. FOPflash is used as the specific control for TOPflash activity. Cells were cultured in 24-well plates until 70-90% confluency. Then, pcDNA3.1, pcDNA3.1-FOXM1, shNC, or shDVL was co-transfected with 0.2 μg of TOPflash plus 10 ng of pRL-SV40 or FOPflash plus 10 ng of pRL-SV40 using Lipofectamine 3000, as indicated. After 48 h, the TOPflash and FOPflash luciferase activity were examined using a dual-luciferase reporter assay system (Promega Corporation, Madison, WI, USA). The luciferase activity of each sample was normalized against Renilla reporter pRL-SV40 (Promega Corporation, Madison, WI, USA) luciferase activity for monitoring transfection efficiency.

### Coimmunoprecipitation (Co-IP)

The protein extracts were incubated with 2 μg of anti-FOXM1 or anti-DVL2 antibody overnight at 4° C. Protein A+G agarose beads were added into the mixture for 6 h of incubation at 4° C. The Bound proteins were collected by centrifuging at 3,000 × g for 5 min at 4° C, and separated from the beads by boiling in sample buffer for 10 min. Subsequently, Western blotting analysis was carried out. The immunoprecipitates and fractions were subjected to Western blotting using antibody as indicated. IgG was used as negative control. The fusion proteins HA-FOXM1 and Flag-DVL2 were immunoprecipitated and examined by incubation with anti-HA and anti-Flag antibody.

### Statistical analysis

Results from three independent experiments were expressed as the mean ± standard deviation (SD). Statistical significance was determined by a two-tailed t-test for comparisons between two groups. A one-way ANOVA was employed to evaluate the differences between groups. All statistical analysis was carried out using GraphPad Prism Software Version 5.0 (GraphPad Software Inc., La Jolla, CA, USA). A value of *P <* 0.05 was regarded as statistically significant.
